# Epidemiology of early childhood weight change and concurrent undernutrition among African children: A sub-continental observational analysis

**DOI:** 10.1371/journal.pone.0339368

**Published:** 2025-12-29

**Authors:** Abiodun Adanikin, Elizabeth Eberechi Oyenusi, Adeniyi Fagbamigbe, Iretiola Fajolu

**Affiliations:** 1 Centre for Healthcare and Communities, Coventry University, United Kingdom; 2 Department of Paediatrics, College of Medicine, University of Lagos, Lagos, Nigeria; University of Petra (UOP), JORDAN

## Abstract

**Background:**

Evidence on weight change among children under five in sub-Saharan Africa is limited, as is knowledge of its association with undernutrition status. This study assessed how early childhood weight change—categorised as acceleration, deceleration, or stable—following different birthweights (low, normal, or high) influences the risk of undernutrition in sub-Saharan African children.

**Methods:**

Using data from 111,623 children across 33 sub-Saharan countries from recent Demographic and Health Surveys (2011–2022), weight trajectory was measured by SD score comparison for current age and birthweight. Survey-weighted logistic regression was used to estimate the odds of distinct weight changes, and of stunting, underweight, and wasting. We adjusted for individual child factors (e.g., age, sex, birth order), maternal factors (e.g., age, education, employment), household factors (e.g., family size, household wealth), and contextual factors (e.g., place of residence).

**Results:**

Among the 111,623 under-five children, 24.63% had accelerated weight change, 40.84% had decelerated weight change, 26.05% were stunted, 11.80% were underweight, and 5.54% were wasted. Of the 38,343 children with ‘stable’ weight progression, 20.14% were stunted, 5.49% were underweight, and 3.27% wasted. The odds of accelerated weight gain were approximately seventeen times higher among low birthweight infants (aOR = 16.92; 95% CI: 15.78 to 18.83), while the odds of decelerated weight change were about nineteen times higher among high birthweight infants (aOR = 18.53; 95% CI: 16.44 to 20.88), compared with those of normal birthweight. Children with normal birthweight who experienced decelerated weight change had highest odds of being underweight (aOR: 14.05; 95% CI: 12.72 to 15.52), followed by LBW infants with accelerated weight gain (aOR: 4.01; 95% CI: 3.48 to 4.62), and high birthweight infants with weight deceleration (aOR: 3.61; 95% CI: 3.11 to 4.17).

**Conclusions:**

Early childhood growth disruptions, especially weight deceleration, are common in sub-Saharan African children. The complex relationship between birthweight, subsequent weight progression, and undernutrition underscores the need to address poverty to prevent growth disruptions and ensure comprehensive growth assessments during healthcare contact by infants to identify suboptimal growth and provide necessary support to address it.

## Background

The physical development of a child, including their weight at a specific age, reflects their accumulated growth experiences [1]. Research has shown that intrauterine environment and nutrition can have a lasting impact on a child’s growth [2,3]. Birthweight may have an inverse programming effect on growth in early childhood, with small-for-gestational-age infants experiencing catch-up growth. Catch-up growth is an abnormally high growth acceleration that occurs in young children after the initial cause of growth deficit is removed [4,5]. It often occurs within the first two years of life, [5], but can extend for a longer period of time in low- and middle-income country settings [6]. In contrast, catch-down growth is a return to the normal physiological growth curve following antenatal growth beyond the genetic potential [7].

The trajectory of growth in early life can have a long-lasting influence. While rapid weight gain for children born small might be beneficial for short-term outcomes such as survival, it has been linked to various medical conditions in adulthood, including cardiovascular diseases, type 2 diabetes, asthma, and obesity [8–11]. However, it has been suggested that in low- and middle-income countries (LMICs), where a substantial number of infants are born small due to poverty and inadequate nutrition, the experience of rapid weight gain and its consequential negative long-term effects may not necessarily be comparable to those observed in high-income countries. Rather, in LMICs, rapid growth may be vital for promoting long-term health and human capital, such as schooling attainment and Intelligence Quotient (IQ) [12–14].

Undernutrition accounts for almost half of deaths among children under five years of age [15]. The prevalence of undernutrition is particularly alarming in sub-Saharan Africa, where 58·7 million children under 5 are stunted and 13·8 million are wasted [16]. Children who are wasted have weakened immune systems and are more susceptible to infections and mortality [17,18], while stunting has been linked to poor intellectual performance [12,14,19]. The uptake of child growth monitoring by households in sub-Saharan Africa is generally poor. Around a third of households in Rwanda and Ethiopia are said to utilise child growth-monitoring services [20,21]. In addition, essential parameters, such as weight and height/length, are not always measured simultaneously. When only changes in weight can be assessed, their effectiveness in predicting undernutrition status, including stunting, remains unclear, as does the relationship between birthweight, subsequent weight progression, and undernutrition. Identifying the relationship between distinct changes in weight over time and undernutrition status in sub-Saharan African children, as well as understanding the factors that contribute to these patterns, will provide valuable information for early childhood assessments and monitoring in low-resource settings.

Therefore, the aim of this research is to evaluate the relationship between early childhood weight change and undernutrition status. We investigated the prevalence and determinants of three distinct weight progression patterns among under-five children, namely “stable”, “acceleration” and “deceleration”, based on changes in the standard deviation (SD) score for weight from birth to the current age, and explored the relationship between these weight patterns and concurrent undernutrition (stunting, underweight and wasting) in sub-Saharan African children. Moreover, we explored the consequences of different weight progression patterns following a low, normal, or high birthweight on the probability of a child being stunted, underweight, or wasted. To our knowledge, this study represents the first attempt to examine and shed light on this relationship in sub-Saharan African children. This knowledge could offer crucial guidance on assessing and monitoring young children in resource-constrained settings and subsequently enable interventions and support that could promote optimal growth.

## Methods

### Study design and data sources

The study data are from the most recent Demographic and Health Surveys (DHS) conducted between 2011 and 2022 in thirty-three sub-Saharan African countries. The DHS Program is a worldwide project initiated by the United States Agency for International Development (USAID). It collects nationally representative population-based data on health indicators including maternal and child health. The program utilises uniform questionnaires, manuals, and multistage cluster sampling techniques to collect comparable information across countries. Further details on the DHS questionnaire, data and access are available at https://dhsprogram.com/. The approval to use the datasets in this study was obtained from MEASUREDHS, which oversees Demographic and Health Surveys, and no additional ethical clearance was necessary.

### Participants

**Fig 1:**
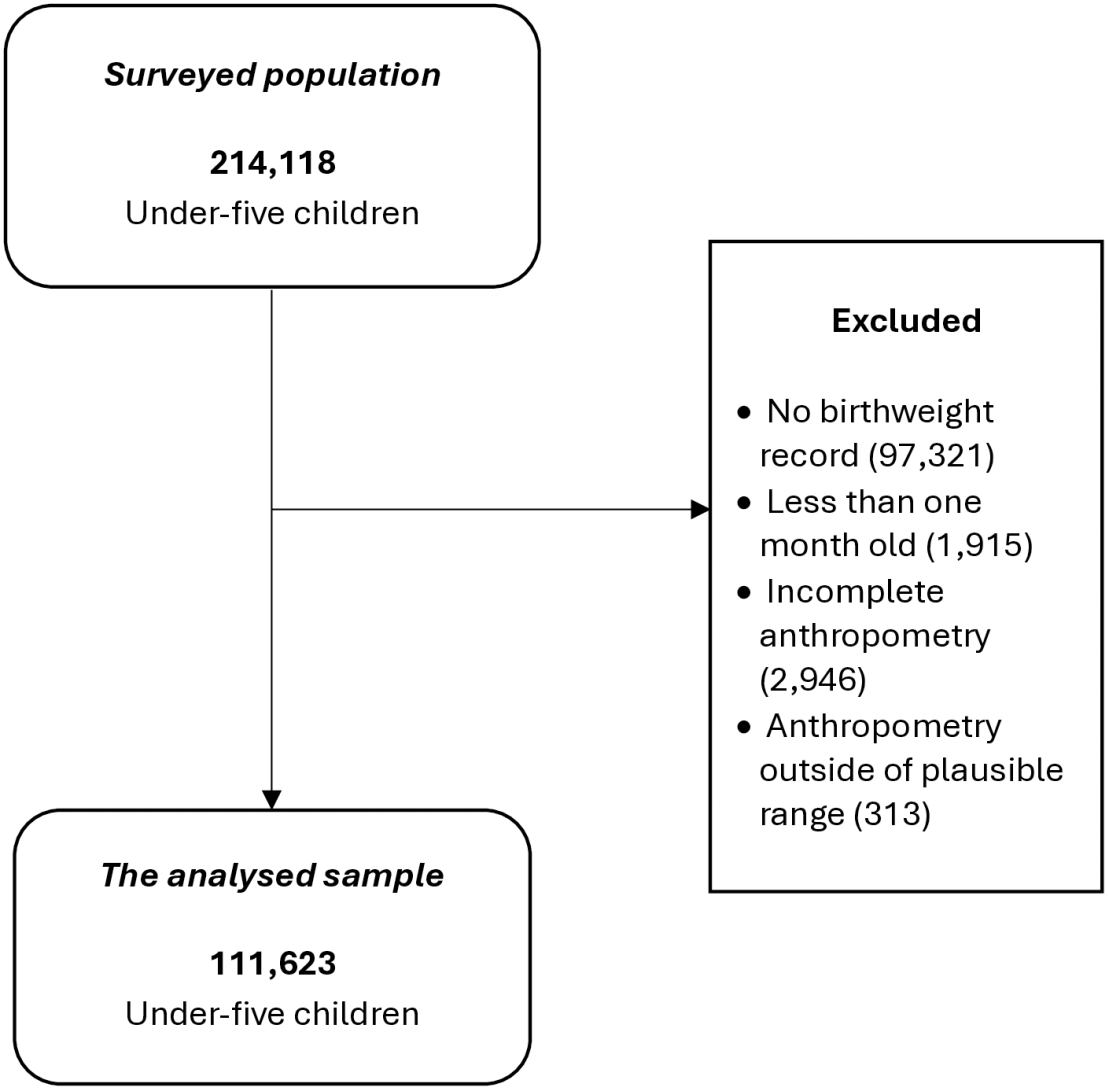
Study population flow chat.

The study population included children under five years of age whose mothers were interviewed and underwent anthropometric measurements during the survey. We excluded children with no birthweight record, who were less than one month old, with incomplete anthropometric data, and measures outside the biologically plausible range, e.g., birthweight < 500g or ≥6500g (Fig 1). We analysed data on 111,623 children from the kids (KR) and household member (PR) files. S1 Table in S1 File displays the contribution of each country in the sub-Saharan African region to the total number of children involved in this study.

### Birthweight data quality

Prior to analysis, we assessed the distribution and quality of birthweight data from the surveys, following the approach proposed by Krasevec et al [22]. Overall, 31% of all birthweights fell on the three most frequently reported values (i.e., 2500g, 3000g, and 3500g); 2.87% were at the extremes (500g and ≥5000g), and 4.73% were ≥ 4500g — indicating no evidence of severe heaping or implausible distribution [22].

### Procedure

#### Outcomes of interest.

The primary outcomes of interest were distinct weight change based on changes in the standard deviation (SD) score for weight between birth and current age and the determining factors. The SD score for weight was determined using the World Health Organization (WHO) 2006 child growth standards corrected for sex and age [23]. The “zscore06” Stata package was used to calculate the z-scores [24]. Weight acceleration was defined as a gain in SD scores exceeding 0·67, whereas weight deceleration referred to a decrease in SD scores exceeding 0·67 [25]. Children with no substantial change in SD scores between the two time points were classified as the “stable” group. The secondary outcome was the relationship between the distinct weight changes and undernutrition (i.e., stunting, wasting and underweight). Stunting was defined as length/height for age z-scores below −2 SD, wasting as weight for height z-scores below −2 SD, and underweight as weight for age z-score below −2 SD [23].

#### Main exposure.

The main exposure was the absolute birthweight in grams, written directly (56% of cases) from the child’s birth card, and maternal recall. Birthweight was categorised as low (< 2500 g), normal (2500–4000 g), and high (> 4000 g) [26–28].

#### Covariates.

We included covariates that have previously been shown to influence early childhood growth trajectories and nutritional status and are available in the DHS [29,30]. We considered individual child factors, including age in months (also representing the interval between birthweight measurement and the DHS anthropometric measurement), sex assigned at birth, birth order, singleton or multiple birth status, and breastfeeding history. Additionally, we considered *maternal factors* such as age, education, employment status, body mass index (BMI), and tobacco use, and *household factors* such as family size and household wealth index – a quintile measure of socioeconomic status, with the lowest quintile representing the “poorest” and the highest quintile representing the “richest”. Furthermore, we adjusted for *contextual factors* such as place of residence and sub-geographical regions of SSA (S2 Table in S1 File).

### Statistical analysis

We applied ‘*svyset*’ command in Stata 18 version and sampling weight to account for the complex survey design and the unequal probability of participants recruitment. We described the weighted prevalence of stable, accelerated, and decelerated weight change based on the characteristics of the study population. To predict the odds of “acceleration” or “deceleration” in weight referent to “stable” weight, we implemented survey-weighted logistic regression with country fixed effects in separate analyses using Stata’s *svy: logit*. This method incorporates the sample design to produce accurate point estimates of population parameters and correct standard errors through Taylor linearisation. In addition, the inclusion of country fixed effects controls for confounding by country-level factors and accounts for heterogeneity in country means. However, we also fitted a survey-weighted multilevel mixed-effect logistic model using *svy: melogit* [31,32], as a sensitivity analysis to assess whether the model specification would alter the substantive conclusions. We considered the constituent sub-Saharan African countries in the study and the clusters which are the primary sampling units (PSU) for each country as random variables, since the children were nested within countries and in turn within the PSU, and the correlation assumption was verified using the intraclass correlation coefficient (ICC). Sampling weights were normalised within each country so that their mean equalled one, ensuring comparability across countries.

We report the adjusted odds ratios and 95% confidence intervals from the models. In addition, we estimated the average predicted probabilities (APPs) of the outcome for key exposures. These APPs represent population-average probabilities adjusted for all model covariates, providing an interpretable measure of absolute risk differences across groups. The two-sided level of statistical significance was set at p < 0·05. This study was conducted in accordance with the Strengthening the Reporting of Observational Studies in Epidemiology (STROBE) guidelines.

### Additional analysis

We conducted stratified analysis to explore the effect modification of current child’s age (0–11 months, 12–23 months, and 24–59 months) on early childhood weight change. To investigate the relationship between distinct weight change patterns and undernutrition status (stunting, wasting, and underweight), we performed separate survey-weighted logistic regression analyses. To explore whether the magnitude of association between weight change and undernutrition status differs based on absolute birthweight, we performed additional analysis assessing the relationship between “normal birthweight and subsequent deceleration in weight”, “high birthweight and subsequent deceleration in weight”, and “low birthweight and subsequent acceleration in weight” referent to “normal birthweight and stable weight progression” and undernutrition. Furthermore, sensitivity analysis was conducted in a subset of the study population (n = 62,875) that had birthweight directly recorded from the birth card to check for consistency of findings with the main analysis that included maternal recall.

### Missing data

Birthweight was missing for 45.45% of children, with missingness associated with maternal education, household wealth, and rural residence. To reduce potential bias, we examined the patterns of missing data and applied multiple imputation by chained equations, incorporating child-level factors, maternal education, household wealth, contextual variables (including country and cluster identifiers), and auxiliary predictors such as place of birth. Sensitivity analyses were then conducted using the imputed birthweight datasets.

## Results

### Prevalence of weight trajectories and undernutrition in the under-five children

**Fig 2:**
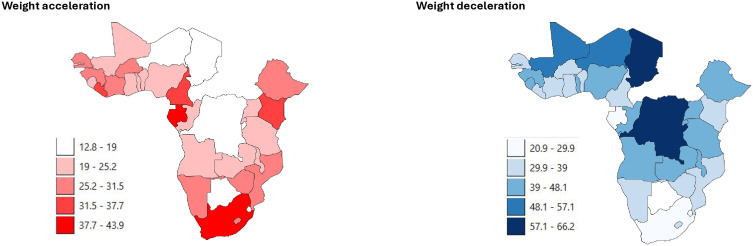
Prevalence of accelerated and decelerated weight change in the sub-Saharan African countries (*Generated from Natural Earth*).

Of the weighted 110,996 under-five children analysed, 10,519 (9·48%) had low birthweight (< 2500 g), and 8,201 (7·39%) had high birthweight (> 4000 g). In total, 27,340 children (24·63%) experienced accelerated weight change, while 45,331 (40·84%) experienced decelerated weight change. Fig 2 shows the prevalence of accelerated and decelerated weight changes in each country within the SSA region. Of the 10,519 children with low birthweight, 8,326 (79·15%) had experienced accelerated change in weight by current age. A total of 37,383 (40·51%) NBW and 7,597 (92·65%) HBW babies demonstrated a deceleration in weight by current age.

A higher proportion of accelerated weight gain was observed in first-born infants, multiple births, children with teenage mothers, and those whose mothers had secondary school education or higher (Table 1). The percentage of children with decelerated weight was lower with increasing maternal BMI and household wealth index.

**Table 1:** Basic description of the study population (weighted frequency).

Variables	Total frequency	Weight change		
		Stable	Acceleration	Deceleration
**Birthweight (grams)**				
LBW (<2500g)	10,519 (9.48)	1,842 (17.51)	8,326 (79.15)	351 (3.33)
NBW (2500 – 4000g)	92,277 (83.14)	35,959 (38.97)	18,935 (20.52)	37,383 (40.51)
HBW (>4000g)	8,201 (7.39)	523 (6.38)	80 (0.97)	7,597 (92.65)
**Current child’s age (months)**				
0-11	25,957 (23.39)	9,151 (35.25)	9,514 (36.65)	7,292 (28.09)
12-23	26,141 (23.55)	9,243 (35.36)	5,944 (22.74)	10,954 (41.90)
24-35	22,872 (20.61)	8,008 (35.01)	4,980 (21.77)	9,885 (43.22)
36-47	19,018 (17.13)	6,446 (33.89)	3,708 (19.50)	8,863 (46.61)
48-59	17,008 (15.32)	5,477 (32.21)	3,194 (18.78)	8,336 (49.02)
**Assigned sex at birth**				
Male	56,112 (50.55)	19,419 (34.61)	13,155 (23.44)	23,539 (41.95)
Female	54,884 (49.45)	18,906 (34.45)	14,185 (25.85)	21,793 (39.71)
**Birth order**				
1^st^	27,462 (24.74)	9,837 (35.82)	8,326 (30.32)	9,299 (33.86)
2^nd^ – 4^th^	56,437 (50.85)	19,734 (34.97)	13,562 (24.03)	23,140 (41.00)
5^th^ and above	27,098 (24.41)	8,754 (32.30)	5,452 (20.12)	12,892 (47.58)
**Plurality of birth**				
Singleton	107,186 (96.57)	37,244 (34.75)	25,482 (23.77)	44,460 (41.48)
Multiple	3,811 (3.43)	1,081 (28.37)	1,858 (48.77)	871 (22.86)
**Ever breastfed**				
Yes	107,050 (96.44)	37,000 (34.56)	26,286 (24.55)	43,764 (40.88)
No	3,946 (3.56)	1,325 (33.56)	1,055 (26.72)	1,567 (39.71)
**Maternal age (years)**				
<20	6,751 (6.08)	2,333 (34.55)	2,259 (33.47)	2,159 (31.98)
20-34	78,919 (71.10)	27,413 (34.74)	19,640 (24.89)	31,866 (40.38)
35-49	25,327 (22.82)	8,580 (33.88)	5,441 (21.48)	11,307 (44.64)
**Maternal education**				
None	28,060 (25.28)	9,502 (33.86)	6,163 (21.96)	12,396 (44.18)
Primary	40,168 (36.19)	13,407 (33.38)	8,917 (22.20)	17,843 (44.42)
Secondary & higher	42,768 (38.53)	15,416 (36.04)	12,260 (28.67)	15,092 (35.29)
**Maternal BMI (kg/m**^**2**^)				
<18.5	6,198 (5.58)	2,007 (32.39)	1,206 (19.46)	2,984 (48.15)
≥18.5 to ≤24.9	56,158 (50.59)	19,036 (33.90)	13,183 (23.48)	23,938 (42.63)
≥25 to ≤29.9	18,475 (16.64)	6,563 (35.53)	4,892 (26.48)	7,020 (38.00)
≥30	9,371 (8.44)	3,394 (36.22)	2,657 (28.36)	3,319 (35.42)
Not known	20,795 (18.73)	7,323 (35.22)	5,402 (25.98)	8,070 (38.81)
**Family size**				
≤4	29,755 (26.81)	10,544 (35.43)	7,969 (26.78)	11,242 (37.78)
≥5	81,241 (73.19)	27,781 (34.20)	19,371 (23.84)	34,089 (41.96)
**Wealth index**				
Poorest	18,060 (16.27)	5,969 (33.05)	3,935 (21.79)	8,155 (45.15)
Poorer	20,355 (18.34)	6,896 (33.88)	4,603 (22.61)	8,857 (43.51)
Middle	22,104 (19.91)	7,499 (33.93)	5,253 (23.76)	9,352 (42.31)
Richer	24,762 (22.31)	8,577 (34.64)	6,310 (25.48)	9,875 (39.88)
Richest	25,716 (23.17)	9,384 (36.49)	7,239 (28.15)	9,093 (35.36)
**Place of residence**				
Urban	48,177 (43.40)	17,381 (36.08)	13,377 (27.77)	17,419 (36.16)
Rural	62,820 (56.60)	20,944 (33.34)	13,963 (22.23)	27,912 (44.43)

Abbreviation: SSA, sub-Saharan Africa; BMI, Body mass index.

A total of 28,912 (26·05%) children under the age of five were stunted, 13,094 (11·80%) underweight, and 6,152 (5·54%) were wasted. Of the 38,343 children with ‘stable’ weight over time, 7,720 (20·14%) were stunted, 2,104 (5·49%) were underweight and 1,255 (3·27%) were wasted. Among the 27,340 children that experienced an acceleration in weight, 3,858 (14·11%) were stunted, 807 (2·95%) were underweight and 549 (2·01%) were wasted. From the 45,331 children that had a deceleration in weight, 17,333 (38·24%) were stunted, 10,183 (22·46%) were underweight and 4,348 (9·59%) were wasted.

### Predictors of weight trajectory

After adjusting for confounding factors, the odds of accelerated weight gain were approximately seventeen times higher (aOR: 16·92; 95% CI: 15·78 to 18·83) in under-five children with LBW compared to NBW babies. Children who are multiple births (aOR: 1·41; 95% CI: 1·23 to 1·61), those who had mothers with secondary or higher-level education (aOR: 1·10; 95% CI: 1·04 to 1·16), those with overweight (OR: 1·21; 95% CI: 1·14 to 1·29) or obese mothers (OR: 1·28; 95% CI: 1·18 to 1·39), and those from the richer (aOR: 1·11; 95% CI: 1·04 to 1·18) or richest households (aOR: 1·33; 95% CI: 1·23 to 1·44) had higher odds of accelerated weight gain. The likelihood of accelerated weight gain decreased with child’s age, birth order, and among those from the poorest households (Table 2). After accounting for other covariates, the model predicted a probability of 78·15% (95% CI: 77·13 to 79·18) for accelerated weight gain among children born with low birthweight.

**Table 2:** Survey-weighted logistic models of acceleration in weight change ^a, b^.

Variables	Accelerated weight change	
	Birth card and maternal recall*	Birth card only*
**Birthweight**		
NBW (ref)		
LBW	16.92 (15.78 - 18.13)	12.40 (11.35 - 13.56)
**Current age**	0.98 (0.97 - 0.98)	0.97 (0.97 - 0.98)
**Sex**		
Male (ref)		
Female	1.05 (1.00 - 1.09)	1.09 (1.04 - 1.15)
**Birth order**	0.93 (0.91 - 0.94)	0.92 (0.90 - 0.94)
**Plurality of birth**		
Singleton (ref)		
Multiple	1.41 (1.23 - 1.61)	1.50 (1.27 - 1.76)
**Maternal age (years**		
20–34 (ref)		
<20	1.03 (0.95 - 1.12)	1.09 (0.99 - 1.21)
35-49	1.13 (1.05 - 1.21)	1.10 (1.01 - 1.20)
**Maternal education**		
Primary (ref)		
No education	1.02 (0.95 - 1.08)	1.02 (0.94 - 1.10)
Secondary or higher	1.10 (1.04 - 1.16)	1.08 (1.01 - 1.17)
**Maternal working**		
No (ref)		
Yes	1.00 (0.96 - 1.05)	1.01 (0.96 - 1.08)
**Maternal BMI (kg/m**^**2**^)		
< 18.5 (ref)		
18.5–24.9	0.69 (0.62 - 0.77)	0.67 (0.59 - 0.76)
25–29.9	1.21 (1.14 - 1.29)	1.20 (1.11 - 1.30)
≥ 30	1.28 (1.18 - 1.39)	1.20 (1.07 - 1.33)
Not known	1.14 (1.02 - 1.27)	1.15 (1.01 - 1.31)
**Tobacco use (incl. smoking)**		
No (ref)		
Yes	1.01 (0.87 - 1.17)	1.00 (0.83 - 1.21)
**Ever breastfed**		
Yes (ref)		
No	0.96 (0.86 - 1.09)	0.92 (0.78 - 1.08)
**Family size**		
≤ 4 (ref)		
5 and above	0.98 (0.94 - 1.03)	1.00 (0.94 - 1.07)
**Household wealth index**		
Middle (ref)		
Poorest	0.90 (0.84 - 0.96)	0.88 (0.80 - 0.96)
Poorer	0.94 (0.88 - 1.01)	0.94 (0.86 - 1.02)
Richer	1.11 (1.04 - 1.18)	1.08 (1.00 - 1.18)
Richest	1.33 (1.23 - 1.44)	1.33 (1.21 - 1.47)
**Place of residence**		
Urban (ref)		
Rural	0.99 (0.93 - 1.04)	1.00 (0.93 - 1.07)

^a^ - “Stable” weight (outcome reference); ^b^ – country fixed effects. Adjusted OR, 95% confidence intervals in brackets.

* Source of birthweight; NBW, normal birthweight; LBW, low birthweight; BMI, body mass index.

In contrast, under-five children with HBW were approximately nineteen times (aOR: 18·53; 95% CI: 16·44 to 20·88) more likely to have a decelerated change in weight than NBW babies. In addition, the odds of a deceleration in weight increased with child’s age, higher birth order, among those from the poorest households, and those residing in rural areas (Table 3). Secondary or higher maternal education reduced the likelihood of weight deceleration (aOR: 0·88; 95% CI: 0·83 to 0·92). High birthweight children had a model-predicted probability of 91·63% (95% CI: 90·73 to 92·52) for decelerated weight change, after accounting for other covariates.

**Table 3:** Survey-weighted logistic models of deceleration in weight change ^a, b^.

Variables	Decelerated weight change	
	Birth card and maternal recall*	Birth card only*
**Birthweight**		
NBW (ref)		
HBW	18.53 (16.44 - 20.88)	15.96 (13.34 - 19.08)
**Current age**	1.02 (1.02 - 1.02)	1.02 (1.02 - 1.02)
**Sex**		
Male (ref)		
Female	0.99 (0.96 - 1.02)	0.95 (0.91 - 0.99)
**Birth order**	1.07 (1.06 - 1.08)	1.08 (1.07 - 1.10)
**Plurality of birth**		
Singleton (ref)		
Multiple	0.71 (0.61 - 0.81)	0.64 (0.54 - 0.78)
**Maternal age (years**		
20–34 (ref)		
<20	0.95 (0.87 - 1.03)	0.88 (0.80 - 0.97)
35-49	0.90 (0.85 - 0.95)	0.91 (0.85 - 0.98)
**Maternal education**		
Primary (ref)		
No education	0.96 (0.91 - 1.01)	0.97 (0.90 - 1.03)
Secondary or higher	0.88 (0.83 - 0.92)	0.91 (0.85 - 0.97)
**Maternal working**		
No (ref)		
Yes	1.01 (0.97 - 1.05)	0.98 (0.93 - 1.04)
**Maternal BMI (kg/m**^**2**^)		
< 18.5 (ref)		
18.5–24.9	1.32 (1.22 - 1.43)	1.33 (1.20 - 1.47)
25–29.9	0.84 (0.79 - 0.88)	0.84 (0.78 - 0.91)
≥ 30	0.77 (0.72 - 0.84)	0.83 (0.75 - 0.92)
Not known	0.93 (0.85 - 1.03)	0.89 (0.79 - 1.00)
**Tobacco use (incl. smoking)**		
No (ref)		
Yes	0.98 (0.87 - 1.10)	1.03 (0.87 - 1.21)
**Ever breastfed**		
Yes (ref)		
No	1.03 (0.93 - 1.14)	1.07 (0.95 - 1.22)
**Family size**		
≤ 4 (ref)		
5 and above	1.02 (0.97 - 1.06)	1.00 (0.94 - 1.06)
**Household wealth index**		
Middle (ref)		
Poorest	1.10 (1.04 - 1.17)	1.11 (1.03 - 1.20)
Poorer	1.01 (0.96 - 1.07)	1.03 (0.96 - 1.10)
Richer	0.94 (0.88 - 0.99)	0.95 (0.88 - 1.02)
Richest	0.75 (0.70 - 0.81)	0.80 (0.73 - 0.87)
**Place of residence**		
Urban (ref)		
Rural	1.08 (1.03 - 1.14)	1.10 (1.03 - 1.17)

^a^ - “Stable” weight (outcome reference); ^b^ – country fixed effects. Adjusted OR, 95% confidence intervals in brackets.

* Source of birthweight; NBW, normal birthweight; HBW, high birthweight; BMI, body mass index.

The sensitivity analysis involving the subpopulation with birthweights obtained directly from the birth card only was consistent with the main analysis (Tables 2 and 3). In this subpopulation, the odds of accelerated change in weight were more than twelve times higher (aOR: 12·40; 95% CI: 11·35 to 13·56) in LBW infants, and the odds of a deceleration in weight were approximately fifteen times greater (aOR: 15·96; 95% CI: 13·34 to 19·08) in HBW infants compared to NBW infants. The results from the survey-weighted multilevel mixed-effects logistic model were also comparable (S3 and S4 Tables in S1 File), indicating that our findings are robust to model specification. Approximately 3.62% and 3.68% of the total variance in accelerated and decelerated weight change, respectively, are attributable to differences between countries. In the stratified analysis by age group, the results were consistent with the main analysis, and the associated predictors remained similar. However, the odds of accelerated or decelerated weight change were slightly higher in the older age groups. Furthermore, the sensitivity analysis using the imputed dataset was consistent with the main analysis (S5 and S6 Tables in S1 File).

### Association between weight trajectories and malnutrition

While accelerated weight change was associated with lower odds of undernutrition, decelerated weight change was associated with stunting, underweight, and wasting. Specifically, under-five children who experienced a deceleration in weight were approximately three-times more likely to be stunted (aOR: 2·86; 95% CI: 2·73 to 2·99), about five times more likely to be underweight (aOR: 5·01; 95% CI: 4·68 to 5·38), and approximately five times more likely to be wasted (aOR: 4·87; 95% CI: 4·45 to 5·33) compared to the ‘stable’ weight group. The highest odds of undernutrition, including stunting, underweight, and wasting, were found in children with normal birthweight who later experienced a weight deceleration. They were particularly at risk of being underweight (aOR: 14·05; 95% CI: 12·72 to 15·52). This was followed by LBW babies that later experienced accelerated change in weight (Table 4).

**Table 4:** Survey-weighted logistic models of undernutrition status (stunting, underweight and wasting) based on early childhood weight change ^a, b^.

Variables	Stunting	Wasting	Underweight
*Weight change*			
Stable (ref)			
Acceleration	0.32 (0.30 - 0.34)	0.23 (0.20 - 0.27)	0.49 (0.44 - 0.55)
Deceleration	2.86 (2.73 - 2.99)	4.87 (4.45 - 5.33)	5.01 (4.68 - 5.38)
*Birthweight & subsequent weight change*			
NBW & Stable weight (ref)			
NBW & weight deceleration	2.93 (2.79 - 3.07)	5.28 (4.77 - 5.86)	14.05 (12.72 - 15.52)
HBW & weight deceleration	1.30 (1.19 - 1.42)	1.67 (1.38 - 2.01)	3.61 (3.11 - 4.17)
LBW & weight acceleration	1.84 (1.70 - 1.98)	1.56 (1.32 - 1.85)	4.01 (3.48 - 4.62)

^a^– multiple survey-weighted logistic regressions summarized in one table; ^b^ – country fixed effects; Model adjusted for child factors (age, assigned sex at birth, birth order, breastfeeding history, and singleton/multiple births), maternal factors (age, education, occupation, BMI, and tobacco use), household factors (family size and wealth index), and place of residence (urban/rural). Results presented as adjusted OR, 95% confidence intervals in brackets.

NBW, normal birthweight, LBW, low birthweight, HBW, high birthweight

## Discussion

We found that approximately a quarter of under-five children in SSA exhibited accelerated weight change, while two-fifths experienced decelerated weight change. The associations of birthweight, child’s age, birth order, maternal education, household wealth, and rural residence were evident in relation to patterns of weight progression. Both initial birthweight and subsequent weight progression were strongly associated with undernutrition status, with substantial decelerated weight change showing significant associations with stunting, underweight, and wasting.

The observation that approximately 75% of the children experienced changes in their weight progression over time is consistent with previous research [33,34]. It is common for a large proportion of infants to express their genetic potential by moving up or down in growth centiles [35]. While HBW infants showed a declining trend in weight during early childhood and LBW infants showed an upward progression, other studies have reported different results [36,37]. It has been postulated that insufficient or excessive nutrition during fetal development can have lasting effects on a child’s appetite and metabolism, leading to issues such as childhood obesity in HBW infants [36,38]. We observed that the LBW and HBW children exhibited accelerated and decelerated weight changes, respectively. However, infants with normal birthweights who might not otherwise be subjected to close early childhood monitoring but who later experience a decline in weight progression are at the greatest risk of undernutrition. While it is widely acknowledged that LBW infants are at an increased risk of undernutrition, our research finding suggests that even normal-to-high birthweight infants in SSA are at risk of stunting, underweight and wasting when exposed to inadequate nutrition and sub-optimal weight growth. It is important to note that LBW infants who experience accelerated weight change are not exempt from stunting, underweight, or wasting. This underscores the need to closely monitor all children, regardless of their birthweight, and accurately assess all anthropometric measures, including weight and height/length, during a child’s contact with health services, address poverty, and implement lifestyle and healthcare measures to promote healthy birthweights in SSA and prevent disrupted growth patterns.

Our research indicates that the first year of life is crucial for reaching genetic growth potential, as the proportion with decelerated weight change increases afterwards. This aligns with prior research [39], and is not unexpected given the high poverty and inadequate nutrition in low-resource settings such as SSA. Our findings emphasise the need for a comprehensive program to monitor the health and development of SSA children until the age five. The position of children relative to their siblings affects their weight progression. Firstborns tend to have higher weights and heights than later-borns by age four, as found in a Brazilian study [40]. Healthcare professionals should continually counsel parents on the benefits of having a suitable number of children who can be adequately nourished and cared for.

Maternal overweight and obesity were found to be associated with accelerated weight change in SSA. Research conducted in developed nations has shown similar outcomes [41,42], suggesting that maternal genetics may affect a child’s weight gain after birth. As observed in this study, secondary or higher maternal education was significantly associated with accelerated weight change in under five children. Although few studies assessing the relationship between maternal education and accelerated weight change have all been conducted in high-income countries [43], a non-significant increased association with accelerated weight change has been noted [44]. The differences in the research context may have contributed to our finding of a statistically significant relationship, indicating that maternal education could influence weight progression in SSA children. Moreover, our findings indicate that household poverty and rural residence, where there may be limited access to child assessment, monitoring and support, may contribute to a decline in weight over time among children from these settings.

This study utilised high-quality, nationally representative, cross-sectional survey data from 33 SSA countries to generate contemporary evidence on early childhood weight change and its relationship with undernutrition among under-five children. The extensive scope of this research, encompassing a substantial sample of children, provided sufficient statistical power to identify meaningful effects. The consistency between the main analysis, which included direct recording of birthweight from the birth card and maternal recall, and the sensitivity analysis, which focused on a subset of participants whose birthweight was directly obtained from the birth card alone, further confirmed the reliability of the findings. It suggests that maternal recall does not substantially bias the associations observed and supports the use of recalled birthweight in analyses when official records (e.g., birth cards) are unavailable. It also provides empirical support for the continued use of maternal recall in large-scale surveys like the DHS, especially in low- and middle-income countries where birth records may be incomplete.

However, this study also had limitations, such as missing data inherent to the utilisation of secondary data sources. Only children with complete anthropometric measurements were included in the main analysis. Birthweight data displayed a systematic pattern of missingness: children of mothers with no education, from the poorest households, and in rural areas were less likely to have a recorded birthweight and were therefore under-represented among complete cases. To address this, we used multiple imputation based on observed predictors (including maternal education, household wealth, and rural residence), which reduces bias under the assumption of missing at random. However, if missingness was influenced by unmeasured factors, some residual bias may persist, with a likely underestimation of poorer outcomes among the most disadvantaged children. Unfortunately, information regarding children’s length at birth was not recorded, precluding the simultaneous evaluation of their linear growth in conjunction with weight change. Nevertheless, insight into the relationship between weight progression and stunting offers an invaluable understanding of the linear growth pattern of the cohort. Due to the lack of gestational age data, we were unable to compute gestational-age-adjusted birthweight z-scores, which limited our ability to differentiate between preterm appropriate-for-gestational-age (AGA) and term small-for-gestational-age (SGA) infants. As a result, it remains unclear whether observed accelerated weight gain in early childhood represents catch-up growth from prematurity or from intrauterine growth restriction. Nevertheless, the findings from this research remain relevant to SSA and similar settings where poor reproductive health literacy, inadequate tracking of the last menstrual period (LMP), and limited access to early pregnancy ultrasounds often result in inaccurate gestational age estimates in practice. We recognise the limitations imposed by the available data to adequately assess the impact of nutrition on the weight progression of the children; however, we endeavoured to capture aspects of child nutrition by accounting for breastfeeding history.

## Conclusion

SSA children experience disrupted growth patterns during their early years, with a more prevalent occurrence of weight deceleration. Growth disruption tends to become more common as children get older. Accelerated weight change was associated with lower odds of undernutrition, while decelerated weight change was associated with higher odds.. Nevertheless, undernutrition remains common among low birthweight infants, even among those with accelerated weight gain. Furthermore, undernutrition was more prevalent among normal birthweight infants who experienced decelerated weight change. The intricate relationship between birthweight, subsequent weight change, and undernutrition in SSA emphasises the significance of tackling poverty, improving maternal education, and precisely assessing all anthropometric measures, such as weight and height/length, when a child encounters healthcare services, in order to detect any potential issues with sub-optimal growth and provide necessary supports to address them.

## Supporting information

S1 File Online supporting information. (DOCX)
