# Supplementary material for: Epidemiology of early childhood weight change and concurrent undernutrition among African children: A sub-continental observational analysis
Source: PLoS One. 2025 Dec 29;20(12):e0339368. doi: 10.1371/journal.pone.0339368 (PMC12747355; doi:10.1371/journal.pone.0339368)
Supplement: S1 File — (DOCX) [file pone.0339368.s001.docx]

**S1 Table. Data contributions from each sub-Saharan African (SSA) country**

| **Country** | **Year of survey** | **Frequency, n (%)** | |
| --- | --- | --- | --- |
|  |  | **Unweighted** | **Weighted** |
| Angola | 2015-16 | 3,274 (2.93) | 3,307 (2.98) |
| Burkina Faso | 2021 | 2,814 (2.52) | 2,787 (2.51) |
| Benin | 2017-18 | 7,413 (6.64) | 7,441 (6.70) |
| Burundi | 2016-17 | 4,802 (4.30) | 4,890 (4.41) |
| Democratic Republic of the Congo | 2013-14 | 5,741 (5.14) | 6,119 (5.51) |
| Republic of the Congo | 2011-12 | 3,762 (3.37) | 3,511 (3.16) |
| Cote d’Ivoire | 2021 | 2,265 (2.03) | 2,124 (1.91) |
| Cameroon | 2018 | 2,961 (2.65) | 3,039 (2.74) |
| Ethiopia | 2016 | 1,807 (1.62) | 1,332 (1.20) |
| Gabon | 2019-21 | 4,717 (4.23) | 4,673 (4.21) |
| Ghana | 2014 | 1,598 (1.43) | 1,622 (1.46) |
| Gambia | 2019-20 | 2,791 (2.50) | 2,740 (2.47) |
| Guinea | 2018 | 1,610 (1.44) | 1,631 (1.47) |
| Kenya | 2022 | 4,240 (3.80) | 4,143 (3.73) |
| Comoros | 2012 | 1,828 (1.64) | 1,911 (1.72) |
| Liberia | 2019-20 | 778 (0.70) | 647 (0.58) |
| Lesotho | 2014 | 1,115 (1.00) | 1,121 (1.01) |
| Mali | 2018 | 3,109 (2.79) | 3,267 (2.94) |
| Malawi | 2015-16 | 4,395 (3.94) | 4,372 (3.94) |
| Mozambique | 2011 | 5,582 (5.00) | 5,330 (4.80) |
| Nigeria | 2018 | 3,194 (2.86) | 3,416 (3.08) |
| Niger | 2012 | 1,498 (1.34) | 1,251 (1.13) |
| Namibia | 2013 | 1,582 (1.42) | 1,475 (1.33) |
| Rwanda | 2019-20 | 3,554 (3.18) | 3,655 (3.29) |
| Sierra Leone | 2019 | 2,986 (2.68) | 2,908 (2.62) |
| Senegal | 2019 | 3,385 (3.03) | 3,276 (2.95) |
| Chad | 2014-15 | 1,053 (0.94) | 1,210 (1.09) |
| Togo | 2013-14 | 1,870 (1.68) | 1,841 (1.66) |
| Tanzania | 2022 | 10,920 (9.78) | 10,925 (9.84) |
| Uganda | 2016 | 2,889 (2.59) | 2,847 (2.56) |
| South Africa | 2016 | 998 (0.89) | 988 (0.89) |
| Zambia | 2018 | 6,860 (6.15) | 6,875 (6.19) |
| Zimbabwe | 2015 | 4,232 (3.79) | 4,321 (3.89) |
| **TOTAL** |  | **111,623 (100)** | **110,996 (100)** |

**S2 Table. Plausible ranges and categorisation of variables**

| **Variables** | **Plausible ranges/categorization** |
| --- | --- |
| Birthweight | Low (<2500g) \| normal (between 2500g and 4000g) \| high (>4000g) |
| Child’s age (in months) | Continuous variable |
| Sex assigned at birth | Male \| Female |
| Birth order | Continuous variable; categorised as: 1^st^ \| 2^nd^ – 4^th^ \| 5^th^ and above |
| Plurality of birth | Single \| Multiple |
| Place of delivery | Public health facility \| Private health facility (incl. mission hospitals) \| Home & other places |
| Ever breastfed | No \| Yes |
| Maternal age | <20 years \| 20-34 years \| 35-49 years |
| Maternal education | None\| Primary \| secondary or higher |
| Mother currently working | No \| Yes |
| Maternal BMI | Underweight < 18.5 kg/m^2^ \| Healthy weight 18.5 – 24.9 kg/m^2^ \| Overweight 25.0 – 29.9 kg/m^2^ \| Obese ≥ 30.0 kg/m^2^ |
| Maternal tobacco use (incl. smoking) | No \| Yes |
| Family size | ≤ 4 \| 5 and above |
| Household wealth index | Poorest \| Poorer \| Middle \| Richer \| Richest |
| Place of residence | Urban \| Rural |
| African subregion | West \| Central \| East \| Southern Africa |

Abbreviation: BMI, Body mass index.

**S3 Table. Survey-weighted mixed effects models of acceleration in weight change. ^a^**

| **VARIABLES** | **Acceleration in weight** |
| --- | --- |
| **Birthweight** |  |
| NBW (ref) |  |
| LBW | 18.64 (15.33 - 22.66) |
| **Current age** | 0.98 (0.97 - 0.98) |
| **Sex** |  |
| Male (ref) |  |
| Female | 1.05 (1.00 - 1.10) |
| **Birth order** | 0.93 (0.91 - 0.94) |
| **Plurality of birth** |  |
| Singleton (ref) |  |
| Multiple | 1.42 (1.20 - 1.69) |
| **Maternal age (years** |  |
| 20 – 34 (ref) |  |
| <20 | 1.03 (0.97 - 1.11) |
| 35-49 | 1.12 (1.04 - 1.21) |
| **Maternal education** |  |
| Primary (ref) |  |
| No education | 1.02 (0.93 - 1.12) |
| Secondary or higher | 1.11 (1.04 - 1.18) |
| **Maternal working** |  |
| No (ref) |  |
| Yes | 1.00 (0.95 - 1.05) |
| **Maternal BMI (kg/m^2^)** |  |
| < 18.5 (ref) |  |
| 18.5 – 24.9 | 0.68 (0.62 - 0.75) |
| 25 – 29.9 | 1.22 (1.15 - 1.29) |
| ≥ 30 | 1.29 (1.18 - 1.41) |
| Not available | 1.15 (1.06 - 1.25) |
| **Tobacco use (incl. smoking)** |  |
| No (ref) |  |
| Yes | 1.01 (0.92 - 1.10) |
| **Ever breastfed** |  |
| Yes (ref) |  |
| No | 0.96 (0.83 - 1.12) |
| **Family size** |  |
| ≤ 4 (ref) |  |
| 5 and above | 0.98 (0.92 - 1.04) |
| **Household wealth index** |  |
| Middle (ref) |  |
| Poorest | 0.90 (0.81 - 0.99) |
| Poorer | 0.96 (0.88 - 1.04) |
| Richer | 1.11 (1.03 - 1.19) |
| Richest | 1.34 (1.25 - 1.43) |
| **Place of residence** |  |
| Urban (ref) |  |
| Rural | 0.97 (0.93 - 1.03) |
| **Random effect** |  |
| SSA Country | 1.14 (1.05 - 1.24) |
| PSU | 1.21 (1.10 - 1.33) |
| ICC% (Country) | 3.62 |
| ICC% (PSU) | 8.84 |

^a^ - “Stable” weight (outcome reference). Adjusted OR, 95% confidence intervals in brackets.

NBW, normal birthweight; LBW, low birthweight; BMI, body mass index; SSA, sub-Saharan Africa; PSU, primary sampling unit; ICC, intraclass correlation coefficient.

**S4** **Table.** **Survey-weighted mixed effects models of deceleration in weight change.** **^a^**

| **VARIABLES** | **Deceleration in weight** |
| --- | --- |
| **Birthweight** |  |
| NBW (ref) |  |
| HBW | 19.95 (16.47 - 24.16) |
| **Current age** | 1.02 (1.02 - 1.03) |
| **Sex** |  |
| Male (ref) |  |
| Female | 0.99 (0.95 - 1.03) |
| **Birth order** | 1.07 (1.06 - 1.09) |
| **Plurality of birth** |  |
| Singleton (ref) |  |
| Multiple | 0.70 (0.59 - 0.82) |
| **Maternal age (years** |  |
| 20 – 34 (ref) |  |
| <20 | 0.94 (0.86 - 1.04) |
| 35-49 | 0.90 (0.84 - 0.95) |
| **Maternal education** |  |
| Primary (ref) |  |
| No education | 0.96 (0.89 - 1.02) |
| Secondary or higher | 0.87 (0.83 - 0.92) |
| **Maternal working** |  |
| No (ref) |  |
| Yes | 1.01 (0.96 - 1.06) |
| **Maternal BMI (kg/m^2^)** |  |
| < 18.5 (ref) |  |
| 18.5 – 24.9 | 1.33 (1.21 - 1.45) |
| 25 – 29.9 | 0.84 (0.80 - 0.88) |
| ≥ 30 | 0.78 (0.72 - 0.84) |
| Not available | 0.93 (0.86 - 1.00) |
| **Tobacco use (incl. smoking)** |  |
| No (ref) |  |
| Yes | 0.98 (0.90 - 1.07) |
| **Ever breastfed** |  |
| Yes (ref) |  |
| No | 1.03 (0.92 - 1.14) |
| **Family size** |  |
| ≤ 4 (ref) |  |
| 5 and above | 1.02 (0.97 - 1.08) |
| **Household wealth index** |  |
| Middle (ref) |  |
| Poorest | 1.10 (1.03 - 1.18) |
| Poorer | 1.01 (0.95 - 1.07) |
| Richer | 0.93 (0.87 - 1.00) |
| Richest | 0.75 (0.69 - 0.82) |
| **Place of residence** |  |
| Urban (ref) |  |
| Rural | 1.09 (1.03 - 1.15) |
| **Random effect** |  |
| SSA Country | 1.14 (1.06 - 1.23) |
| PSU | 1.19 (1.09 - 1.31) |
| ICC% (Country) | 3.68 |
| ICC% (PSU) | 8.53 |

^a^ - “Stable” weight (outcome reference). Adjusted OR, 95% confidence intervals in brackets.

NBW, normal birthweight; HBW, high birthweight; BMI, body mass index; SSA, sub-Saharan Africa; PSU, primary sampling unit; ICC, intraclass correlation coefficient.

**S5 Table**. **Survey-weighted logistic models of acceleration in weight change (imputed).** **^a, b^**

| **VARIABLES** | **Acceleration in weight** |
| --- | --- |
| **Birthweight** |  |
| NBW (ref) |  |
| LBW | 20.23 (19.01-21.53) |
| **Current age** | 1.00 (0.99-1.00) |
| **Sex** |  |
| Male (ref) |  |
| Female | 0.97 (0.94-1.00) |
| **Birth order** | 1.01 (1.00-1.02) |
| **Plurality of birth** |  |
| Singleton (ref) |  |
| Multiple | 1.06 (0.95-1.19) |
| **Maternal age (years** |  |
| 20 – 34 (ref) |  |
| <20 | 1.12 (1.05-1.20) |
| 35-49 | 0.92 (0.88-0.96) |
| **Maternal education** |  |
| Primary (ref) |  |
| No education | 1.55 (1.48-1.62) |
| Secondary or higher | 0.85 (0.81-0.89) |
| **Maternal working** |  |
| No (ref) |  |
| Yes | 0.94 (0.90-0.98) |
| **Maternal BMI (kg/m^2^)** |  |
| < 18.5 (ref) |  |
| 18.5 – 24.9 | 0.91 (0.85-0.98) |
| 25 – 29.9 | 0.95 (0.90-1.00) |
| ≥ 30 | 0.99 (0.92-1.06) |
| Not available | 1.53 (1.41-1.66) |
| **Tobacco use (incl. smoking)** |  |
| No (ref) |  |
| Yes | 2.43 (2.19-2.69) |
| **Ever breastfed** |  |
| Yes (ref) |  |
| No | 3.52 (3.25-3.82) |
| **Family size** |  |
| ≤ 4 (ref) |  |
| 5 and above | 1.04 (1.0-1.08) |
| **Household wealth index** |  |
| Middle (ref) |  |
| Poorest | 1.49 (1.41-1.58) |
| Poorer | 1.23 (1.17-1.30) |
| Richer | 0.94 (0.89-0.99) |
| Richest | 0.86 (0.80-0.92) |
| **Place of residence** |  |
| Urban (ref) |  |
| Rural | 1.37 (1.30-1.44) |

^a^ - “Stable” weight (outcome reference); ^b^ – country fixed effects. Adjusted OR, 95% confidence intervals in brackets.

NBW, normal birthweight; LBW, low birthweight; BMI, body mass index; SSA, sub-Saharan Africa.

**S6 Table**. **Survey-weighted logistic models of deceleration in weight change (imputed).** **^a, b^**

| **VARIABLES** | **Deceleration in weight** |
| --- | --- |
| **Birthweight** |  |
| NBW (ref) |  |
| HBW | 20.09 (18.15-22.25) |
| **Current age** | 1.01 (1.01-1.01) |
| **Sex** |  |
| Male (ref) |  |
| Female | 1.03 (1.0-1.06) |
| **Birth order** | 1.02 (1.01-1.04) |
| **Plurality of birth** |  |
| Singleton (ref) |  |
| Multiple | 0.74 (0.65-0.84) |
| **Maternal age (years** |  |
| 20 – 34 (ref) |  |
| <20 | 0.9 (0.83-0.97) |
| 35-49 | 1.01 (0.96-1.06) |
| **Maternal education** |  |
| Primary (ref) |  |
| No education | 0.7 (0.67-0.74) |
| Secondary or higher | 1.0 (0.95-1.05) |
| **Maternal working** |  |
| No (ref) |  |
| Yes | 1.06 (1.02-1.11) |
| **Maternal BMI (kg/m^2^)** |  |
| < 18.5 (ref) |  |
| 18.5 – 24.9 | 1.15 (1.07-1.23) |
| 25 – 29.9 | 0.95 (0.91-1.01) |
| ≥ 30 | 0.89 (0.83-0.96) |
| Not available | 0.75 (0.69-0.82) |
| **Tobacco use (incl. smoking)** |  |
| No (ref) |  |
| Yes | 0.55 (0.49-0.61) |
| **Ever breastfed** |  |
| Yes (ref) |  |
| No | 0.42 (0.38-0.46) |
| **Family size** |  |
| ≤ 4 (ref) |  |
| 5 and above | 0.99 (0.95-1.03) |
| **Household wealth index** |  |
| Middle (ref) |  |
| Poorest | 0.8 (0.75-0.84) |
| Poorer | 0.86 (0.82-0.91) |
| Richer | 1.0 (0.95-1.06) |
| Richest | 0.93 (0.87-0.99) |
| **Place of residence** |  |
| Urban (ref) |  |
| Rural | 0.87 (0.83-0.91) |

^a^ - “Stable” weight (outcome reference); ^b^ – country fixed effects. Adjusted OR, 95% confidence intervals in brackets.

NBW, normal birthweight; HBW, high birthweight; BMI, body mass index; SSA, sub-Saharan Africa.
